# Supplementary material for: A neural circuit model for human sensorimotor timing
Source: Nat Commun. 2020 Aug 7;11:3933. doi: 10.1038/s41467-020-16999-8 (PMC7414125; doi:10.1038/s41467-020-16999-8)
Supplement: Supplementary file 1 — Supplementary Information [file 41467_2020_16999_MOESM1_ESM.pdf]

Supplementary Materials

A neural circuit model for human sensorimotor timing

Seth W. Egger, Nhat M. Le, and Mehrdad Jazayeri

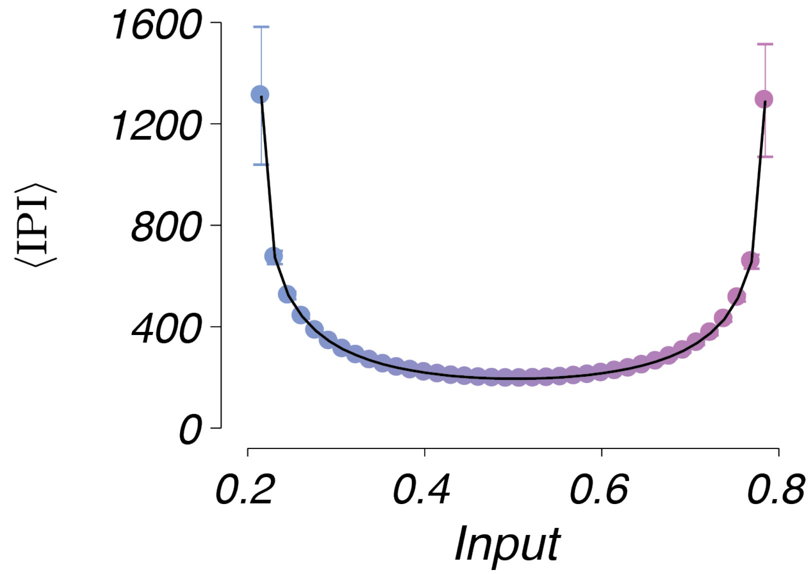

Supplementary Figure 1. Mean inter-production-interval (IPI) as a function of input I. We simulated the MPM with I ranging from 0.2 to 0.8 (colors) and $\sigma_n = 0.01$. For $I < 0.212$ and $I > 0.788$, the dynamics of u and v are constrained such that their difference is always less than y_0 and therefore cannot drive y to threshold. For $0.212 < I < 0.5$, the mean IPI is monotonically decreasing. For $0.5 < I < 0.788$, the mean IPI is monotonically increasing.

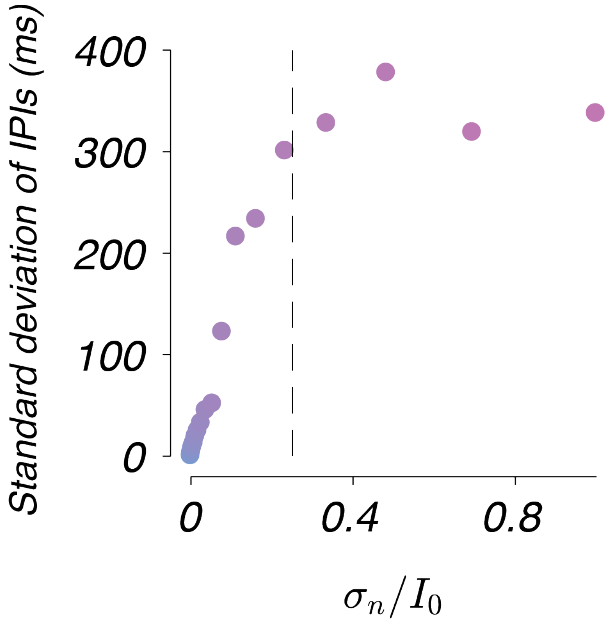

Supplementary Figure 2. Standard deviation of IPI of the MPM increases with noise level. We simulated the MPM at different levels of σ_n and I_0 (colors). Results for $I_0 = 0.76$ are shown. For σ_n less than 25% of I_0 (vertical dashed line), noise increased monotonically with σ_n. After that point, the dynamics of the MPM became dominated by the noise and variability in the IPI saturates.

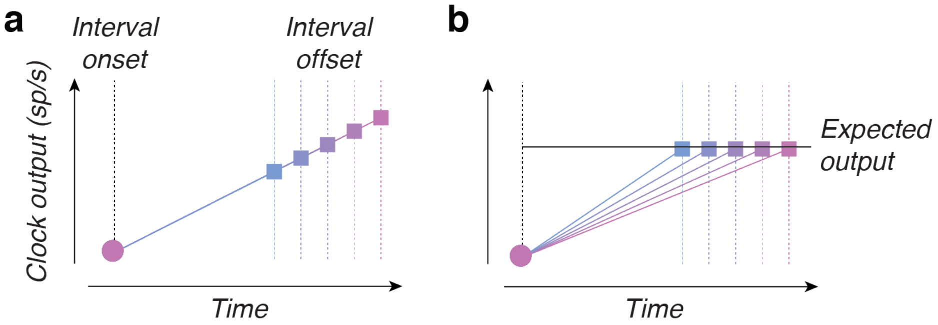

Supplementary Figure 3. Absolute and predictive timing mechanisms. a) Absolute timing mechanism. In this mechanism, neural systems integrate the ticks from a central clock, generating activity that increases monotonically (colored solid lines) from interval onset (black dashed line) to interval offset (colored dashed lines). Because the rate of increase does not depend on the interval, the output level serves as a continuous estimate of the elapsed time, and the level of activity at the time of the interval offset (squares) signals interval duration. Colors correspond to different interval durations. b) Predictive timing mechanism. In this mechanism, the rate of increase is adjusted so that the output reaches the same point after an anticipated interval (vertical dashed lines). If the rate of increase is set correctly, the output at the time of interval offset (squares) will match the expected output (black horizontal line). Output that is less than the expectation indicates the interval is shorter than anticipated while output that is larger than the expectation indicates the interval is longer than anticipated. Colors as in panel a.

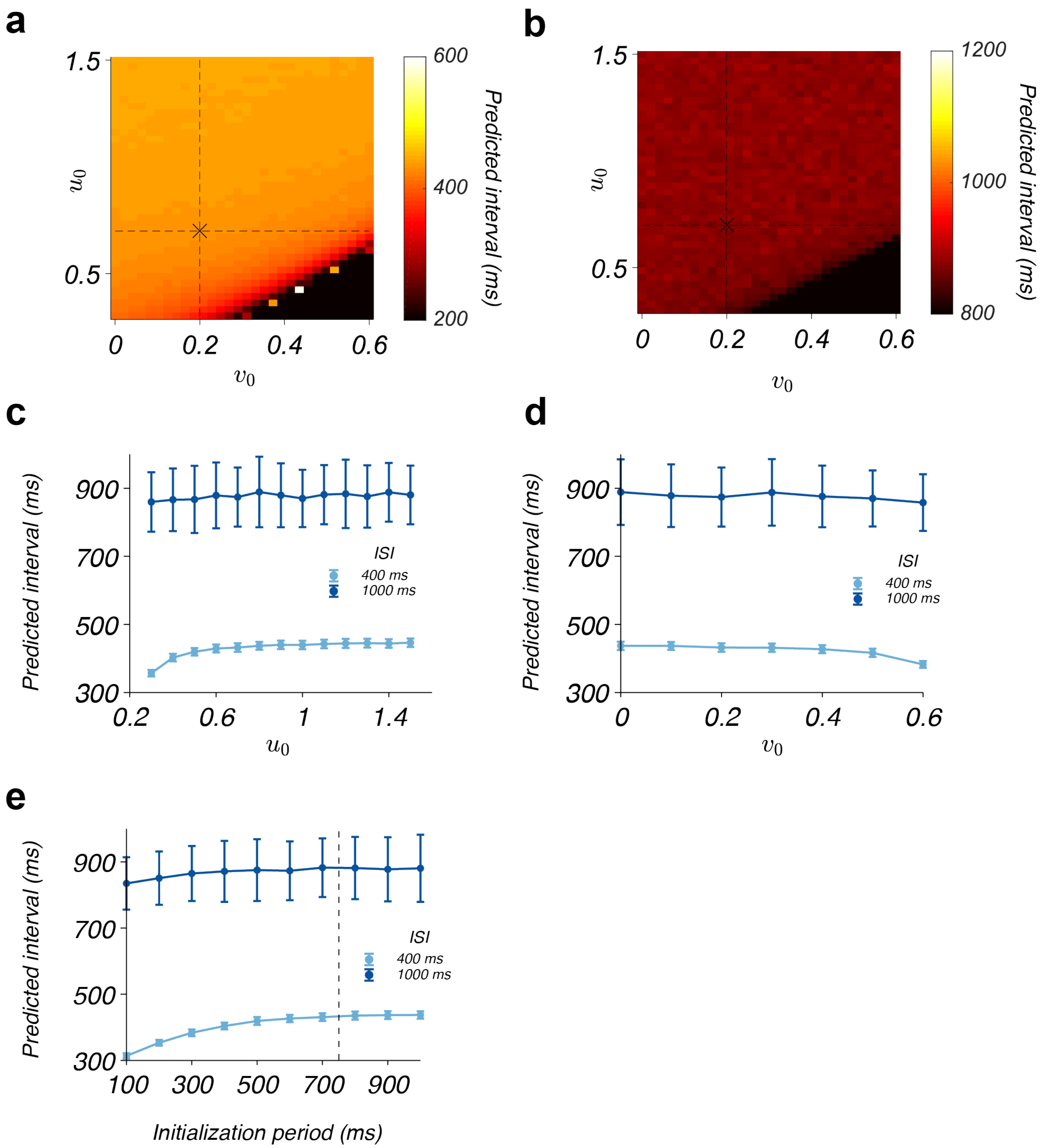

Supplementary Figure 4. Dependence of SAM performance on initial conditions. We simulated the SAM using the same set of parameters as Figure 3b ($K = 5, I_0 = 0.77, \sigma_n = 0.01$) during the presentation of three beats of an isochronous rhythm. a) Mean predicted interval (the interval between the last beat and when y_s crosses y_0) as a function of the initial states (u_0 and v_0) for an inter-stimulus-interval (ISI) of 400 ms. b) Same as panel a for an ISI of 1000 ms. The cross indicates the initial states used in the main text ($u_0 = 0.7, v_0 = 0.2$). c) Vertical cross-sections of panels a and b at $v_0 = 0.2$. d) Horizontal cross-sections of panels a and b at $u_0 = 0.7$. e) Mean predicted intervals of the SAM ($\pm$ standard deviation) as a function of the initialization period for ISI of 400 ms or 1000 ms. Vertical dashed line indicates the initialization period used in the paper (750 ms). In panels c-e, error bars represent the standard deviation across $n = 300$ simulations.

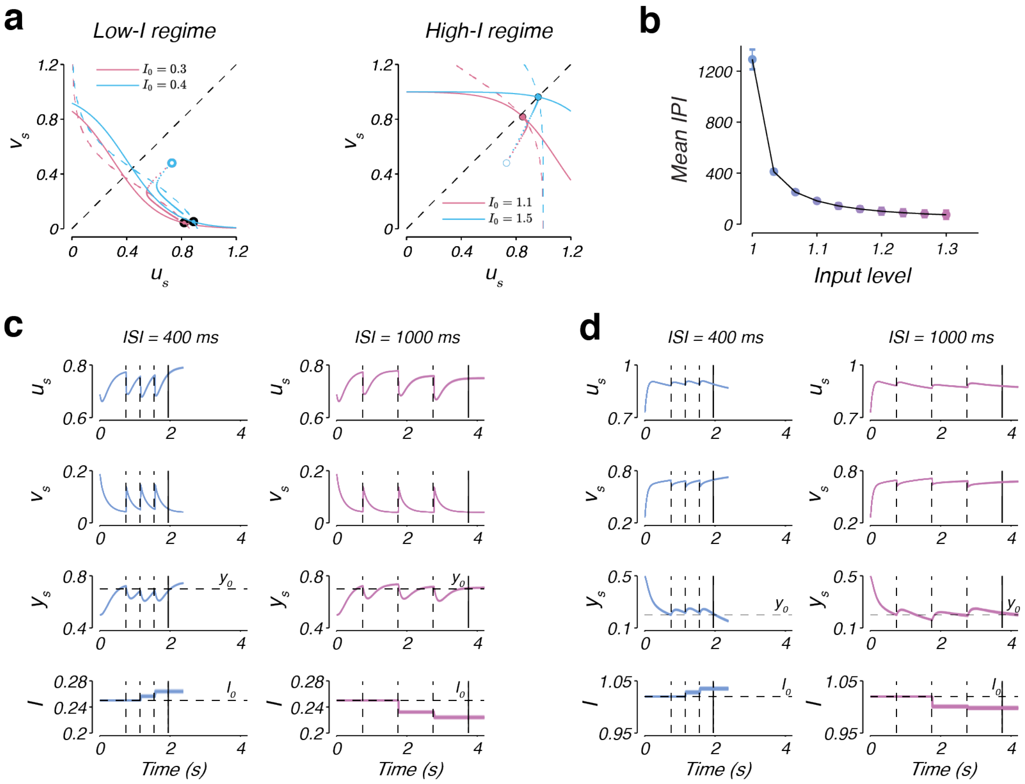

Supplementary Figure 5. Modified SAM for timing anticipation at different input regimes. a) Phase plane of the units u_s and v_s, showing trajectories taken by the system at two input regimes, low-I ($I < 0.5$, left), and high-I ($I > 1.0$, right). Open circles indicate the initial state, and filled circles represent the terminal fixed points of the system at different input levels. b) Mean of the IPIs ($\pm$ standard deviation, across $n = 500$ productions) as a function of the input level, I (colors), of the modified circuit model that operates in the high-I regime. c) Example responses of the SAM units u_s, v_s, y_s and I in the low-I regime to three equidistant stimuli with an ISI of 400 ms (left), or 1000 ms (right). Conventions and model parameters are the same as in Figure 3b. The circuit implementation is identical to our main SAM model, with the exception of the I update, which is reversed in sign, $\tau \frac{dI_s}{dt} = -sK(y_s - y_0)$. Under these conditions, the model generates (1) differences in trajectory speed and (2) parallel neural trajectories, consistent with neural data[1] in a similar task. d) Same as panel c, but for the high-I regime. The circuit implementation is identical to our main SAM model, except the threshold, y_0, was changed to 0.2, and the activity of the unit y_s approaches the threshold from above. Although the circuit successfully matches IPI to the ISI in this regime, it fails to generate parallel neural trajectories in the $u_s - v_s$ plane.

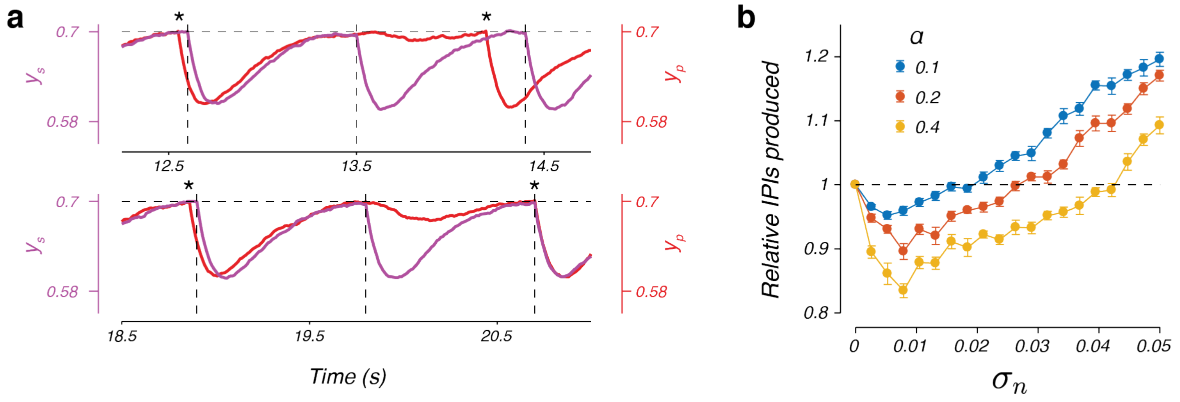

Supplementary Figure 6. Interaction of noise and α results in skipped productions. a) Examples of skipped productions by the full circuit in response to an 800 ms ISI. Lines show the output of the MPM (y_{p}, red) and the SAM (y_{s}, purple) for $\alpha = 0.2$ (top) and $\alpha = 0.4$ (bottom) with $\sigma_{\mathrm{n}} = 0.0079$. Vertical dashed lines indicate the times of the stimuli and asterisks indicate the time at which y_{p} crosses threshold. In each case, noise prevents one threshold crossing before the SAM is reset. b) Relative number of IPIs ($\pm$ standard error) for different values of σ_{n} and α. We performed 10 simulations of the full circuit model with different values of σ_{n} (abscissa) and α (colors) in response to 100 isochronous stimuli (ISI=800 ms), and measured the number of IPIs relative to when $\sigma_{\mathrm{n}} = 0$. For low levels of noise, increasing α results in the circuit missing more productions, as indicated by the decrease in relative number of IPIs produced. As the level of noise increases, the circuit IPI behavior becomes increasingly variable, leading to an increased probability of early productions in addition to skipped productions. As a result, more IPIs are produced when the level of noise is high relative to the circuit without noise.

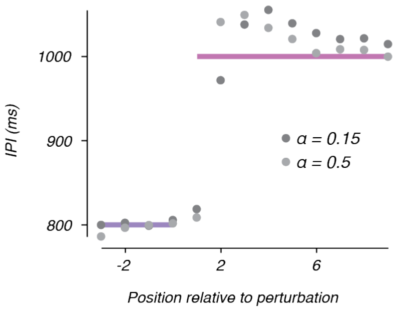

Supplementary Figure 7. Overshoot of circuit model IPIs after a step change in ISI for different levels of α. The degree of IPI overshoot after a step change in ISI from 800 ms (blue line) to 1000 ms (pink line) increases with the level of α. To demonstrate this, we simulated the full circuit, as in Figure 6a, but changed the level of α from 0.15 (dark gray circles) to 0.5 (light gray circles) while keeping K fixed at 3. The larger value of α leads to a larger overshoot early in the response to a step change in the ISI. This effect arises from the circuit attempting to match the phase of the stimuli, which is also perturbed by the step change in ISI. When α is large, the augmented input to the MPM will also be large in amplitude, resulting in an increased response to the phase difference and a longer mean IPI following the step change.

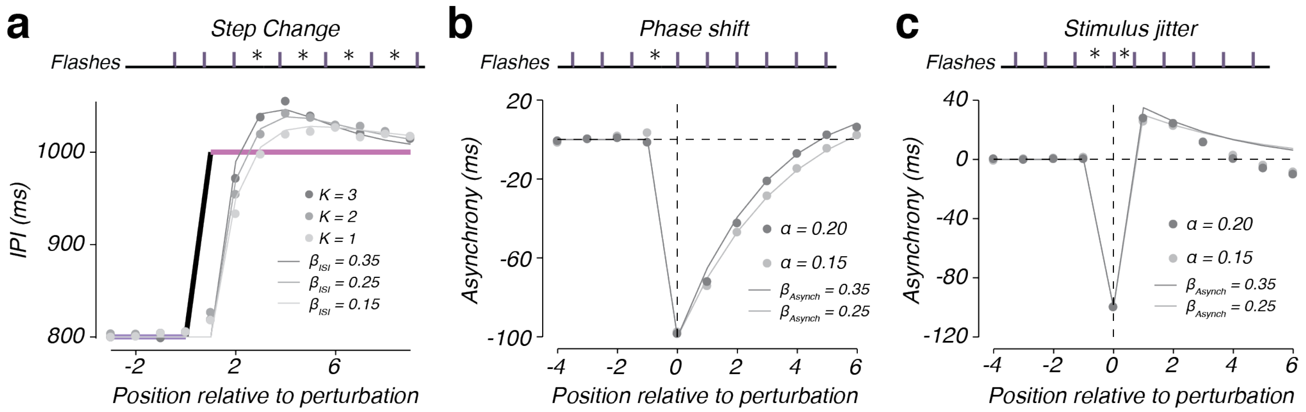

Supplementary Figure 8. Comparison of circuit and algorithmic responses to stimulus perturbations. Following Repp[2], we modeled the time of the t_{n+1}th motor output according to a linear behavioral algorithm (see Methods). Briefly, we set $t_{n+1} = t_n + \beta_{\text{Asynch}} a_n + T_{n+1}$. T_{n+1} represents the anticipated ISI between the nth and $n+1$th stimulus and is calculated according to $T_{n+1} = T_n + \beta_{\text{ISI}}(T_n - \text{ISI}_n)$. a_n represents the asynchrony between the nth stimulus, m_n, and the nth production, t_n, and was calculated according to $a_n = t_n - m_n$. a) Comparison of circuit model (circles) and linear algorithm (lines) in response to a step change in the ISI from 800 ms (blue line) to 1000 ms (pink line). Different levels of gray correspond to different weighting of errors in anticipated flash timing (K and β_{ISI} for the circuit and algorithmic models, respectively). b) Circuit and linear algorithm in response to a phase shift. Conventions as in panel a, but gray scale now corresponds to the sensitivity to phase differences controlled by α for the circuit model and β_{Asynch} for the algorithmic model. c) Circuit and linear algorithm response to stimulus jitter. Conventions as in panel b.

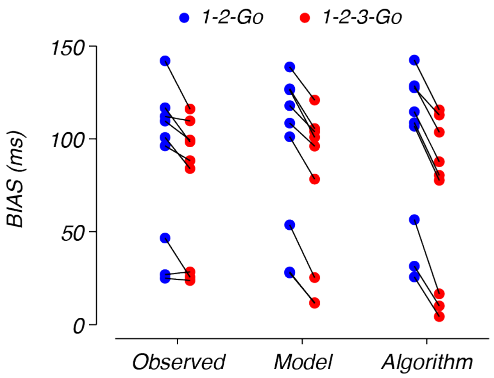

Supplementary Figure 9. Subject and model BIAS in 1-2-Go and 1-2-3-Go. Left: observed subject BIAS (see Methods), middle: BIAS of circuit model, and right: BIAS of linear behavioral algorithm, in 1-2-Go and 1-2-3-Go. Dots represent the data for each condition and lines connect data points across conditions for the same subject.

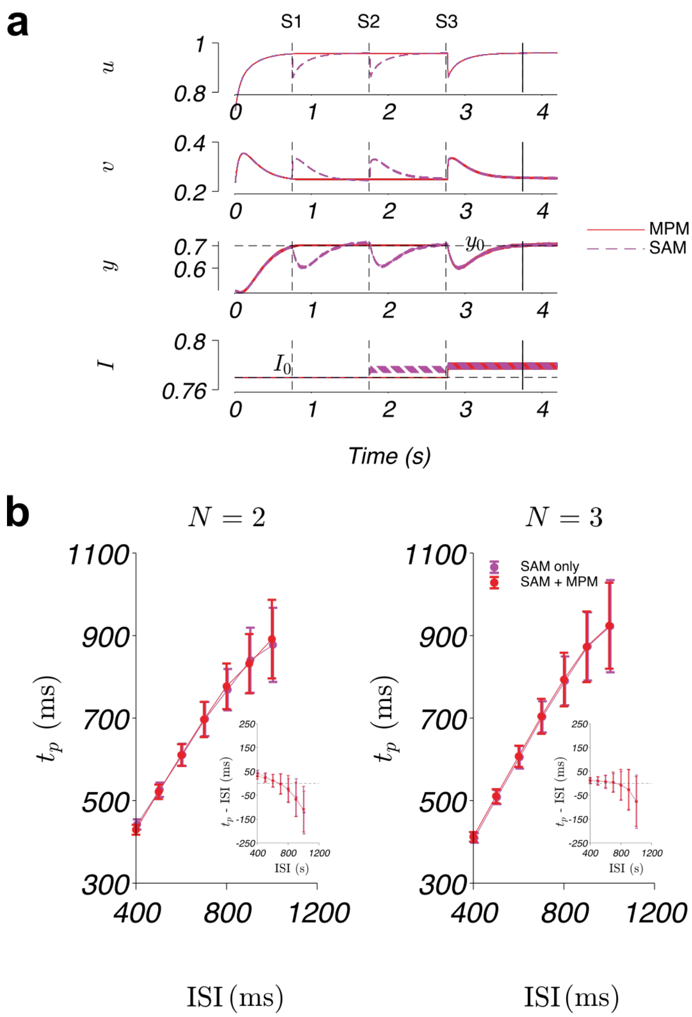

Supplementary Figure 10. Production by the MPM during the interval reproduction task. We considered an alternative mechanism for the interval reproduction task in which both the SAM and MPM are involved. Here, the output of the MPM is suppressed until after the final stimulus is shown by clamping the output of the units u and v at their respective values when threshold y_0 is reached and setting $\alpha = 0$. After the last stimulus, the MPM is driven by the input of the SAM and is responsible for producing the final motor output. a) Response of the units of the MPM (red) and SAM (purple) to three equidistant stimuli with an ISI of 1000 ms. Conventions and model parameters are the same as in Figure 3b. b) Production times t_p (mean $\pm$ standard deviation) by our original model with the SAM only (purple, as in Figure 3b), or the alternative model with the SAM and MPM combined (red), for $N = 2$ stimuli (left) and $N = 3$ stimuli (right). Insets: mean timing difference between production and the expected time of the next stimulus ($\pm$ standard deviation).

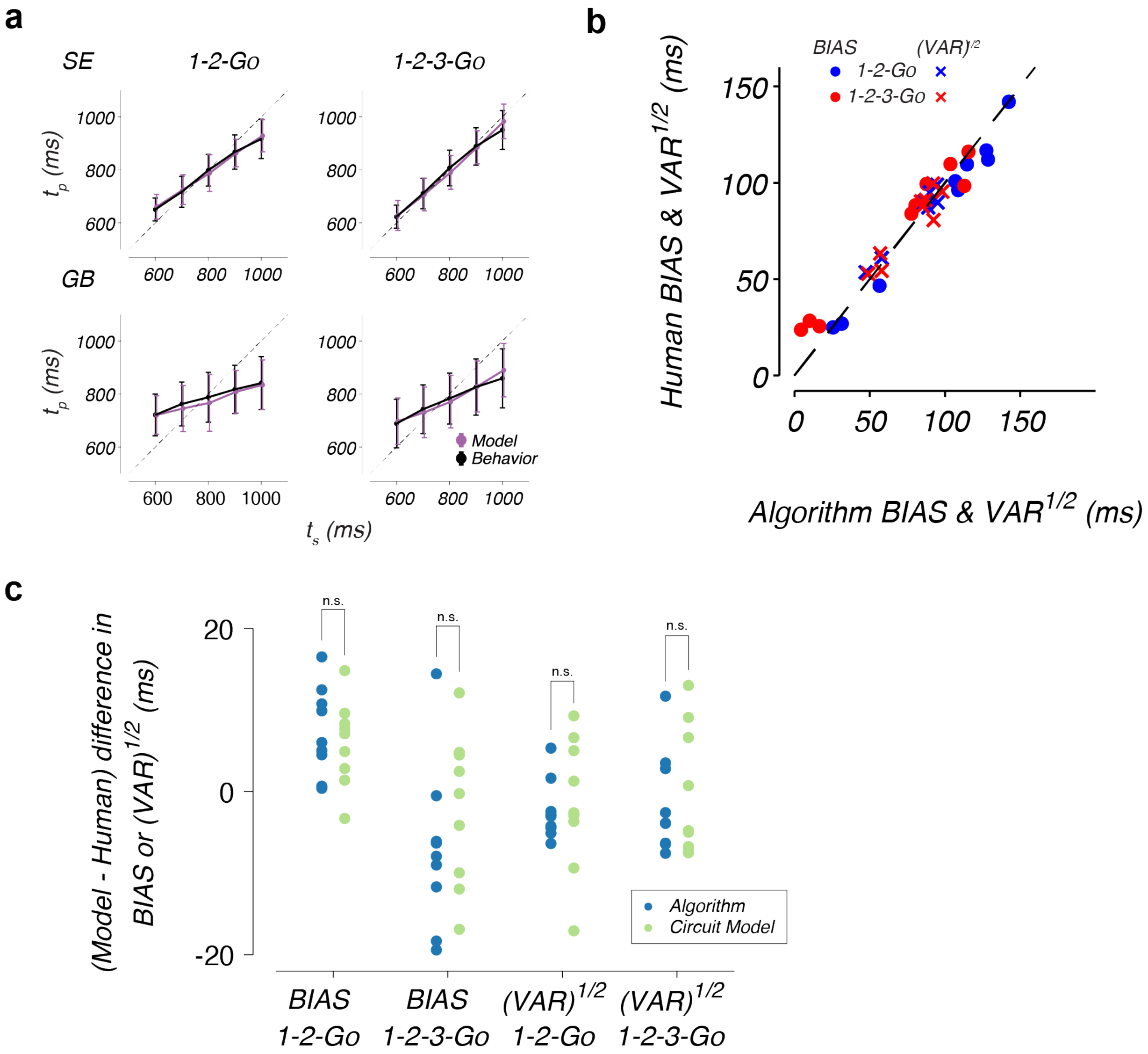

Supplementary Figure 11. Circuit model, linear algorithm, and human behavior in interval reproduction tasks. a) Linear behavioral algorithm fit to example subject behavior (see Methods). Convention is the same as in Figure 7b. Error bars represent standard deviation across trials ($n = 100$ simulated trials for each t_S). b) BIAS and VAR of linear algorithm and subjects. Conventions are the same as in Figure 7c. c) Difference in BIAS and VAR between the two models and human behavior. For each model and each subject, we calculated the discrepancies $\mathrm{BIAS}_{\mathrm{model}} - \mathrm{BIAS}_{\mathrm{subject}}$, or $\mathrm{VAR}^{1/2}_{\mathrm{model}} - \mathrm{VAR}^{1/2}_{\mathrm{subject}}$. These discrepancies are not statistically significantly different between the two models (BIAS in 1-2-Go: $z = 1.066, p = 0.8$; BIAS in 1-2-3-Go: $z = -1.42, p = 0.08$; $\mathrm{VAR}^{1/2}$ in 1-2-Go: $z = -0.237, p = 0.4$; $\mathrm{VAR}^{1/2}$ in 1-2-3-Go: $z = 0, p = 0.5$, two-sided Wilcoxon signed-rank test).

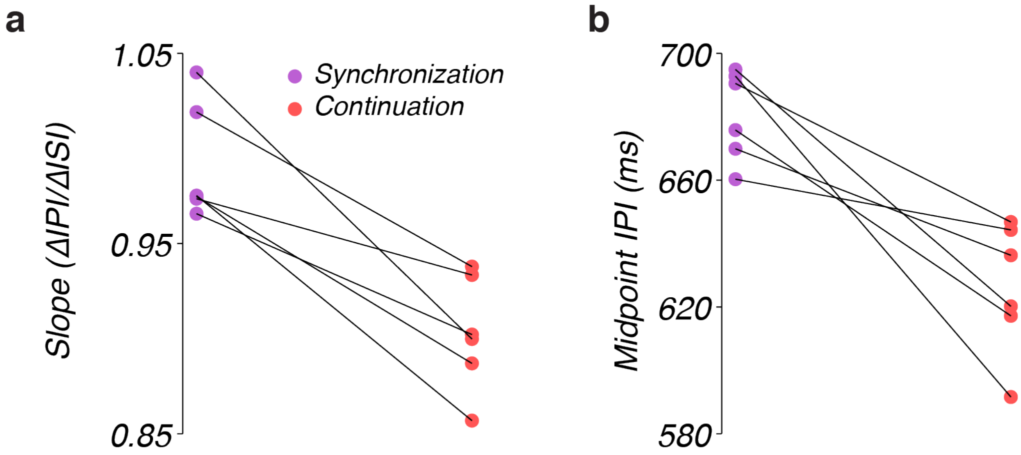

Supplementary Figure 12. Sensitivity of the interproduction interval (IPI) of the circuit model to interstimulus interval (ISI) and the overall offset of the IPI during synchronization and continuation. a) Sensitivity measured as the slope of the regression line fit to IPI versus ISI data during synchronization and continuation. Data points plot the slope of individual subjects and lines connect synchronization and continuation data for each subject. b) Overall offset measured as the mean IPI during synchronization and continuation in response to an ISI of 700 ms. The 700 ms ISI was the midpoint of the prior distribution of ISIs. Therefore, the mean IPI in response to the 700 ms ISI indicates the degree to which the overall IPI response curve is shifted relative to the midpoint of the data.

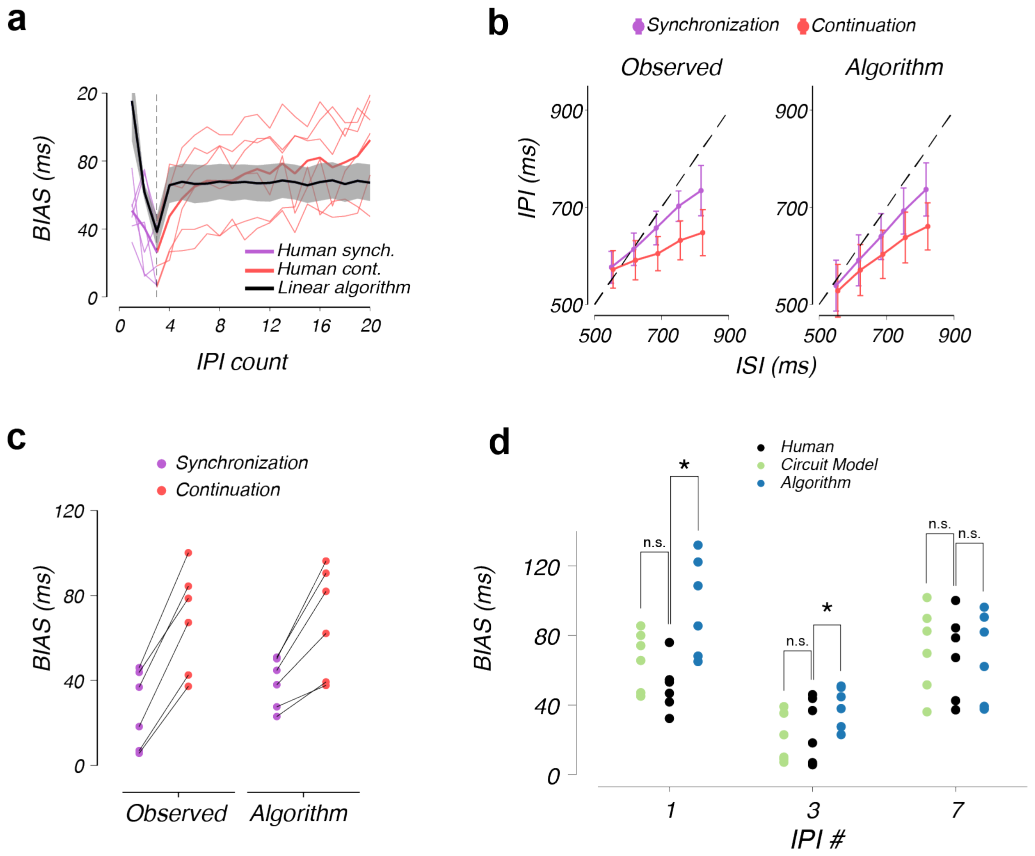

Supplementary Figure 13. Circuit model, linear algorithm, and human behavior in the synchronization/continuation task. In panels a-c, conventions are the same as in Figures 8a-c. a) Overall BIAS in the synchronization/continuation task. b) IPI for different ISIs for an example subject (left) and the linear behavioral algorithm fit to that subject's behavior (right; see Methods). Error bars represent standard deviation across trials, $n = 21$ simulation trials for each ISI. c) Observed and algorithm BIAS for subjects during each phase of the task. d) BIAS of human subjects (black), the circuit model (green), and the linear behavioral algorithm (blue) in the first, third, and seventh IPIs. Each dot represents an individual subject or the model fit to that subject. Note that the first and third IPIs occur during synchronization while the seventh IPI occurs during continuation. For all three IPIs, the BIAS is not statistically different (n.s.) between the circuit model and human behavior (First IPI, $z = 1.78, p = 0.07$, third IPI, $z = -0.52, p = 0.6$, seventh IPI, $z = 1.15, p = 0.2$, two-sided Wilcoxon signed-rank test). In contrast, the BIAS is significantly different between the algorithm and human behavior for the first and third IPIs (asterisks; First IPI, $z = 2.20, p = 0.028$, third IPI, $z = 2.20, p = 0.028$, seventh IPI, $z = -0.10, p = 0.9$, two-sided Wilcoxon signed-rank test). These results demonstrate that the augmented input pathway allows the circuit model to capture the pattern of biases observed in human subjects better than previous models that assumed linear corrections to timing errors.

Subject	σ_n	I_0	K	Anticipated t_s associated with I_0 and σ_n (ms)
SE	0.0085 ± 0.0003	0.7782 ± 0.0006	5.8 ± 0.2	850 ± 50
CV	0.017 ± 0.002	0.7788 ± 0.0005	2.7 ± 0.3	850 ± 90
GB	0.022 ± 0.002	0.7790 ± 0.0004	2.5 ± 0.4	850 ± 100
LB	0.0054 ± 0.0003	0.780 ± 0.001	6.0 ± 0.2	940 ± 50
PG	0.020 ± 0.003	0.7792 ± 0.0003	1.8 ± 0.4	860 ± 100
SM	0.0060 ± 0.0003	0.779 ± 0.001	6.6 ± 0.5	890 ± 50
TA	0.017 ± 0.001	0.7797 ± 0.0008	1.8 ± 0.4	880 ± 100
VD	0.0234 ± 0.004	0.778 ± 0.001	1.7 ± 0.4	800 ± 100
VR	0.0247 ± 0.002	0.7755 ± 0.0006	1.9 ± 0.5	740 ± 90

Supplementary Table 1. Parameters of model fits to the 1-2-Go and 1-2-3-Go behavior (mean ± standard deviation across 5 optimization runs for each subject). The speed of dynamics before sensory feedback corresponded to an ISI of 850.25 ± 39.26 ms, consistent with anticipating the ISI based on an approximation of the prior mean.

Subject	I_0	K	α
AL	0.767 ± 0.002	1.9 ± 0.6	0.08 ± 0.01
ER	0.765 ± 0.003	4.2 ± 0.6	0.077 ± 0.009
FK	0.771 ± 0.002	4.0 ± 0.6	0.05 ± 0.01
KL	0.768 ± 0.002	1.7 ± 0.6	0.08 ± 0.01
MW	0.770 ± 0.003	4.4 ± 0.4	0.055 ± 0.008
RC	0.766 ± 0.002	3.1 ± 0.7	0.09 ± 0.01

Supplementary Table 2. Parameters of model fits to the synchronization/continuation behavior (mean $\pm$ standard deviation across 20 optimization runs for each subject).

References

1. Egger, S. W., Remington, E. D., Chang, C.-J. & Jazayeri, M. Internal models of sensorimotor integration regulate cortical dynamics. *Nat. Neurosci.* **22**, 1871–1882 (2019).

2. Repp, B. H. Sensorimotor synchronization: a review of the tapping literature. *Psychon. Bull. Rev.* **12**, 969–992 (2005).
